# Rational engineering of physicochemical properties of nanomaterials for biomedical applications with nanotoxicological perspectives

**DOI:** 10.1186/s40580-016-0064-z

**Published:** 2016-02-01

**Authors:** P. N. Navya, Hemant Kumar Daima

**Affiliations:** 1 Nano-Bio Interfacial Research Laboratory (NBIRL), Department of Biotechnology, Siddaganga Institute of Technology, Tumkur, 572103 Karnataka India; 2 Amity Institute of Biotechnology, Amity University Rajasthan, Jaipur, 303007 Rajasthan India

**Keywords:** Rational design, Nanomaterials, Nanomedicine, Physicochemical, Nanotoxicology

## Abstract

Innovative engineered nanomaterials are at the leading edge of rapidly emerging fields of nanobiotechnology and nanomedicine. Meticulous synthesis, unique physicochemical properties, manifestation of chemical or biological moieties on the surface of materials make engineered nanostructures suitable for a variety of biomedical applications. Besides, tailored nanomaterials exhibit entirely novel therapeutic applications with better functionality, sensitivity, efficiency and specificity due to their customized unique physicochemical and surface properties. Additionally, such designer made nanomaterials has potential to generate series of interactions with various biological entities including DNA, proteins, membranes, cells and organelles at nano-bio interface. These nano-bio interactions are driven by colloidal forces and predominantly depend on the dynamic physicochemical and surface properties of nanomaterials. Nevertheless, recent development and atomic scale tailoring of various physical, chemical and surface properties of nanomaterials is promising to dictate their interaction in anticipated manner with biological entities for biomedical applications. As a result, rationally designed nanomaterials are in extensive demand for bio-molecular detection and diagnostics, therapeutics, drug and gene delivery, fluorescent labelling, tissue engineering, biochemical sensing and other pharmaceuticals applications. However, toxicity and risk associated with engineered nanomaterials is rather unclear or not well understood; which is gaining considerable attention and the field of nanotoxicology is evolving promptly. Therefore, this review explores current knowledge of articulate engineering of nanomaterials for biomedical applications with special attention on potential toxicological perspectives.

## Introduction

**Fig. 1:**
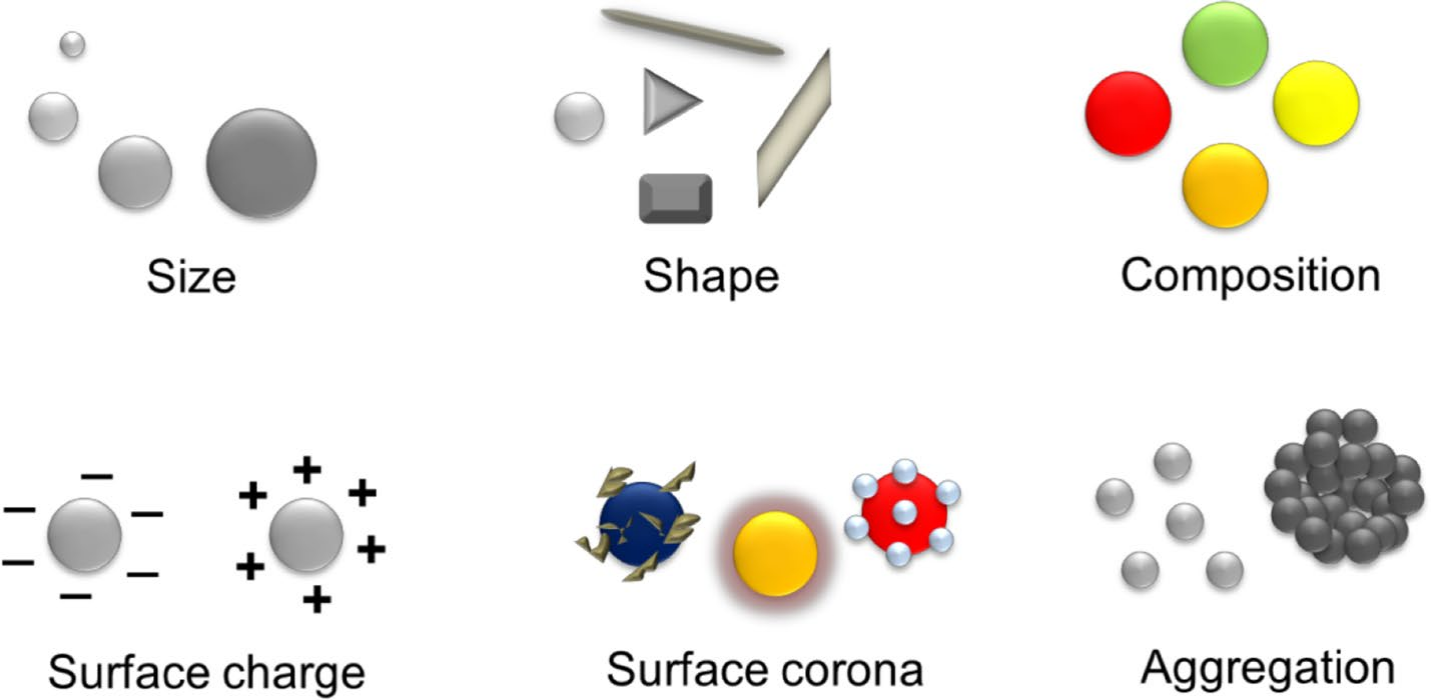
Schematic representation of various physicochemical properties of nanomaterials which influences their biomedical potentials

Nanostructures are engineered assemblies of materials with at least one dimension equivalent to 100 nm or less as defined by the National Nanotechnology Initiative (NNI). These nano scale materials are significantly important and increasingly being employed for commercial purposes in various sectors, wherein some of the advanced nanomaterials are at the leading edge of nascent fields of nanobiotechnology and nanomedicine [1–6]. Material at nano scale range exhibit unique physicochemical properties, which are accredited to their ultra-small size, high surface to volume ratio, composition, presence of biochemical moieties on surface (peripheral coatings or functional groups), hydrophilic or hydrophobic nature, physical appearance (shape or morphology) and aggregation [7] as illustrated in Fig. 1.

Due to the above stated unusual physicochemical belongings, nanomaterials differ considerably from the bulk material of the alike composition, permitting them to execute remarkable feats of better functionality, sensitivity, efficiency and specificity in terms of their therapeutic or biomedical applications [1, 8, 9]. Furthermore, contemporary progress in the field of nanotechnology has given ability to rationally design a variety of nanomaterials and manipulate their chemical, physical and potential biological properties for drug screening (labelling), gene delivery (transfection), diagnosis/monitoring (devices and labelling), drug delivery (therapy), detection (imaging), tissue engineering and other biomedical applications. It is apparent that the nanomedicine is equivalent to traditional medicine but with better prospects to diagnose precisely and promptly, to cure diseases efficiently without or minimal side effects. For example, by manipulating therapeutic agents and other materials at the nanoscale level, their essential properties and bioactivity can be transformed. Such transformed characteristics can permit control over therapeutic agents/drugs in terms of their solubility, blood pool retention times, controlled release over short or long durations, environmentally triggered controlled release or definite site-targeted delivery [1, 4, 5, 8, 10–22].

In the context of nanomedicine, a variety of materials have been utilized for their potential medical applications, wherein metallic nanoparticles have been proven the most convenient and suitable due to their unique optical, physical and electrical properties. These materials have found noteworthy applications in imaging, sensing, drug delivery and gene targeting. Numerous studies related to metallic particles such as gold and silver nanoparticles have been discussed in different sections of this review in terms of their application and toxicity [18, 23–31]. In addition to metallic nanoparticles, carbon based materials such as fullerenes, nanotubes, nanodiamonds and graphene are important nanomaterials for biomedical applications. Fullerenes, graphene and their derivatives have shown good biocompatibility which makes them attractive candidate for biomedical applications especially for bio-sensing, -imaging and drug delivery. Fullerenes have been regarded as a double-edged sword; because they display therapeutic applicability at lower concentrations; however, at the higher concentrations they induce inflammation and if chronic, may promote cancer. Likewise, contemporary research has indicated that graphene and its derivatives may cause cytotoxicity in experimental in vitro and in vivo conditions along with genotoxicity and innovative methodologies need to be employed to evaluate their toxicities [32]. Another carbon based material diamond nanoparticles have shown their importance as single-particle biomarker for fluorescence imaging. Moreover, surface of these nanoparticles can be effectively functionalized to bind with a variety of proteins and nucleic acids, empowering them to be employed as a carrier for pharmaceutical agents or oligonucleotides [21, 33–37].

**Fig. 2:**
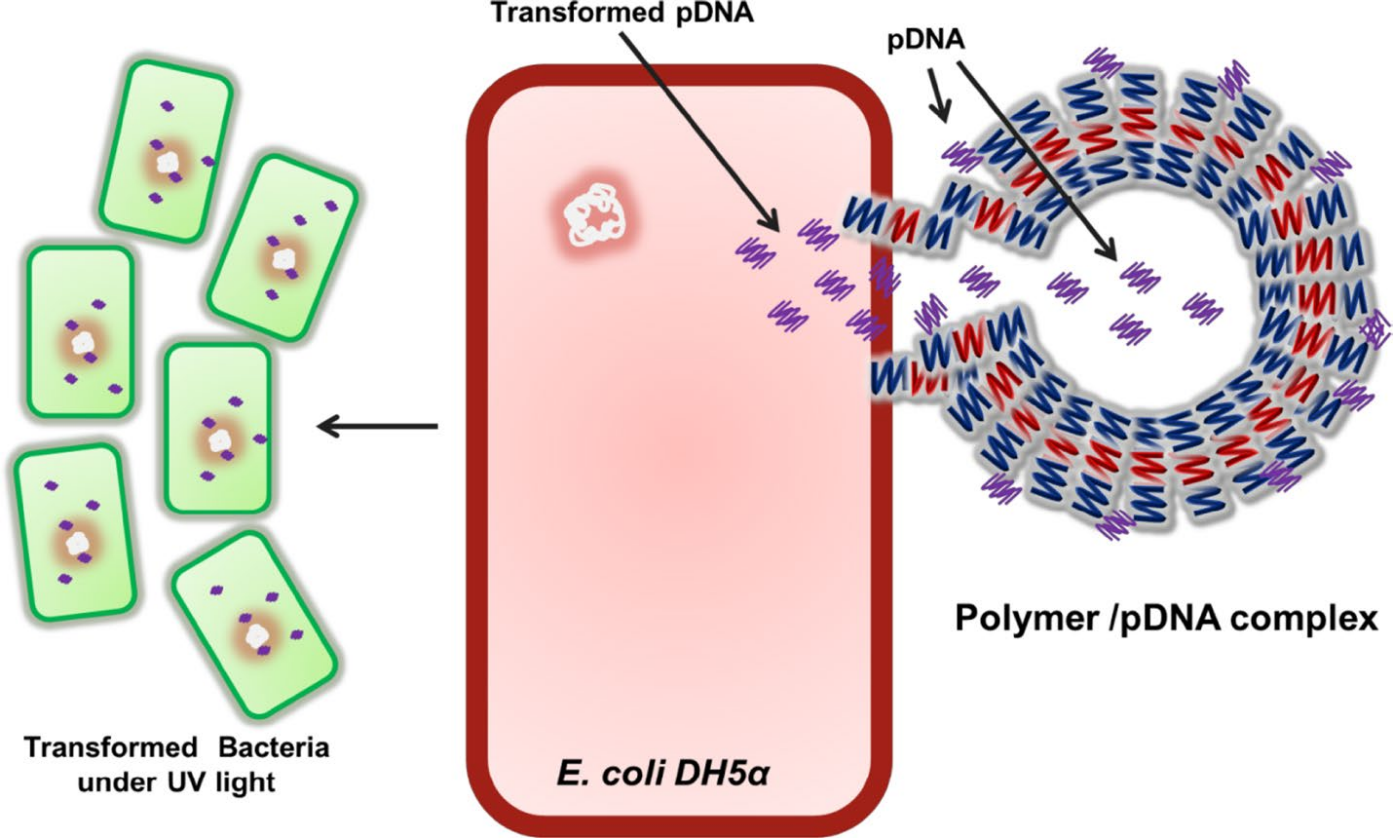
Schematic representation of plasmid DNA delivery in cellular environment by employing copolymer P-123 (PEO_20_–PPO_69_–PEO_20_) as delivery vector

Quantum dots (QDs) have also emerged as a novel class of fluorescent probe for in vivo biomolecular and cellular imaging due to their size-tuneable light emission, improved signal brightness, resistance toward photo-bleaching and simultaneous excitation of multiple fluorescence colours. Moreover, current research has led fabrication of multifunctional nano-probes that are highly bright and stable under different in vivo conditions. Additionally, polymer-encapsulated QDs have been prepared by encapsulating luminescent QDs with amphiphilic block copolymers and linking the polymer coating to tumor-targeting ligands and drug delivery functionalities. Interestingly, these materials have been found to be nontoxic to the cells and such conjugated QDs have raised new possibilities for ultrasensitive and multiplexed imaging of molecular targets in living cells, animal models and possibly in humans; however, their long-term in vivo toxicity and degradation need to be more carefully evaluated [38]. In addition to above discussion, polymeric nanomaterials have also attracted significant interest, which are colloidal structures and composed of synthetic or semisynthetic polymers. These materials have extensive potential for biomedical applications and predominantly being used for drug or gene delivery purposes due to their less toxic properties. The drug moieties can be entrapped, encapsulated or attached to a polymeric matrix for biological applications. [39, 40]. In this context, our group has demonstrated construction of soft nanostructures of biocompatible tri-block copolymer P-123 (PEO_20_–PPO_69_–PEO_20_) [poly(ethylene oxide)–poly(propylene oxide)–poly(ethylene oxide)] for their utility as a non-viral DNA delivery vector in cellular environment using *Escherichia coli* DH5α as a model microorganism. In this research, optimum weight ratio of 1:10 of plasmid DNA to copolymer P-123 was screened to achieve higher transformation efficiency. The schematic mechanism by which pDNA and copolymer P-123 nano-constructs release pDNA into the bacterium has been illustrated in Fig. 2, wherein PEO the hydrophilic part of polymer complex adsorbed on the cell wall and PPO the hydrophobic part can insert into the cell and efficiently deliver pDNA [41]. Another important material at nanoscale level is liposome, that contains a lipid bilayer membrane surrounded by an aqueous interior mimicking the biologic membranes for improving the efficacy and safe delivery of anti-cancer, anti-fungal, antibiotic drugs, anesthetics and anti-inflammatory drugs along with the delivery of gene medicines [42].

Despite of numerous potential biomedical applications, toxicological perspective of engineered nanomaterials is poorly understood or rather unclear, which is gaining considerable attention in terms of nanotoxicology. Although, nanotoxicology is in embryonic stage of its development; it is a vital part of nanomedicine and discusses interactions of engineered nanomaterials with biological systems or environment; wherein, particular emphasis is given on the correlations between the physicochemical and surface properties of nanomaterials with induction of toxic or adversarial biological responses. In addition to this, nanotoxicology aims to discover favourable physicochemical characteristics of various nanomaterials, which may render them more responsive toward inner biological environment for therapeutic benefits [43, 44]. Therefore, the response of active biomolecule with living entity should be more closely related to the quantity of active molecule coming into the direct contact with biological object rather it’s transient initial distribution or administered mass concentration. In a typical nanotoxicity study, engineered nanomaterials are introduced in specialized media for biological application and dose is described as the total particle mass/number, surface area or volume of the particles per unit volume of liquid media or per unit surface area of the well (sedimentation surface). However, in recent past more consideration has been given to the mass transport (sedimentation/diffusion) of particles in suspension, which proceeds at a rate governed by the mass transport properties (sedimentation/diffusion-coefficients) of the formed agglomerates in suspension [45–47]. Therefore, the requirement for precise in vitro dosimetry remains foremost hindrance to the further development of cost-effective toxicological screening methods for engineered nanomaterials to realize their full potential for biomedical applications [48]. Therefore, a careful selection of in vitro doses for nanoparticles toxicity testing is imperative, which largely depend upon the effective density and diameter of formed agglomerates in suspension [49]. From the above discussion, it appears that there is contradiction between nanomedicine and nanotoxicology in terms of application and safety. Therefore, this review aims to explore current knowledge of engineering various physicochemical characteristics of materials at nano scale level for biomedical applications with potential toxicological perspective.

In the context of biomedical applications of nanomaterials, it is vital to recognize that the concomitance of nanomaterials and biological entity may exert detrimental effects on biological systems [50, 51]. These adverse effects are created due to nano-bio interfacial interactions, which are driven by a series of communications between nanomaterial and natural boundaries of biological entities such as DNA, proteins, membranes, cells and organelles. Such interactions are motivated by colloidal forces and depend on vibrant bio-physico-chemical properties of nano-bio boundary leading to form protein corona, particle wrapping, intracellular uptake and bio-catalytic progressions that may be bio-compatible or -adverse in nature [7, 23, 52].

In terms of nanomaterials toxicity, three principles have been elucidated which are referred as transport principle, surface principle and materials principle. All these fundamental principles of materials toxicity need to be considered pragmatically for dictating specific interactions between nano objects and biological systems. Moreover, these three basic principles provide insight of each nanomaterial separately for their specific physicochemical property, which are imperative in creating adversarial biological effects [51].

Therefore, to contrive nanomaterials for biomedical applications, it is imperative to rationally engineer nanomaterials with controlled physicochemical properties to dictate nano-bio interface toward desired interactions to achieve highest level of safety with better functionality, sensitivity, efficiency and specificity. Moreover, basic understanding of nano-bio interfacial interactions between engineered nanomaterials and biological objects will allow predictive relationships at the nano-bio interface. Such predictive interactions are essential for the perspective of further development of designing strategies and safe usage of nanomaterials [53–55]. From the discussion, it can be established that prior to utilizing nanomaterials in the field of medicine or biology, the effects of nanomaterials must be anticipatable and defined, and nanomaterials must exhibit desired therapeutic outcomes without or negligible cytotoxicity.

In this perspective, a variety of nanomaterials have been rationally designed including engineered metallic nanoparticles, their alloys and oxides, super-paramagnetic oxide crystals, quantum dots, semiconductors, dendrimers, polymeric micelles, liposomes, aquasomes (carbohydrate-ceramic nanoparticles) and polyplexes/lipopolyplexes for their vast potential towards biomedicine application from diagnostics to treatment of disease [1, 25, 26, 56–60]. Moreover, it is imperative to state that the dimensional resemblance of designer made nanomaterials and biomolecules (enzymes, DNA, membrane, proteins etc.) provides noteworthy potential to tailored nanomaterials to substantial influence biomedical sciences by achieving desired sensitivity with improved bio-functionality, -efficiency and -specificity as discussed earlier [18, 61–63]. Furthermore, contemporary progressions in meticulous synthesis, development in functionalization strategies and tranquil atomic scale tailoring of physicochemical properties of nanomaterials positioning such materials at forefront for several biomedical applications including biomolecular detection/diagnostics, drug/gene delivery, fluorescent labelling, tissue engineering, biochemical sensing etc. In spite of what we have achieved so far, a complete understanding of how cells interact with well-defined nanomaterial at the molecular level remains poorly understood and more insight need to be provided. Furthermore, it is inevitable to systematically investigate and analyse any unwanted toxicity or risks associated with nanomaterials prior to make a final clinical translation. Therefore, the next section will discuss that how various physicochemical properties of nanomaterials can be rationally engineered to influence their biomedical potential to achieve desired biological goal without any toxicological impact.

## Engineering physicochemical properties at nanoscale for biomedical applications with controlled nanotoxicity

**Fig. 3:**
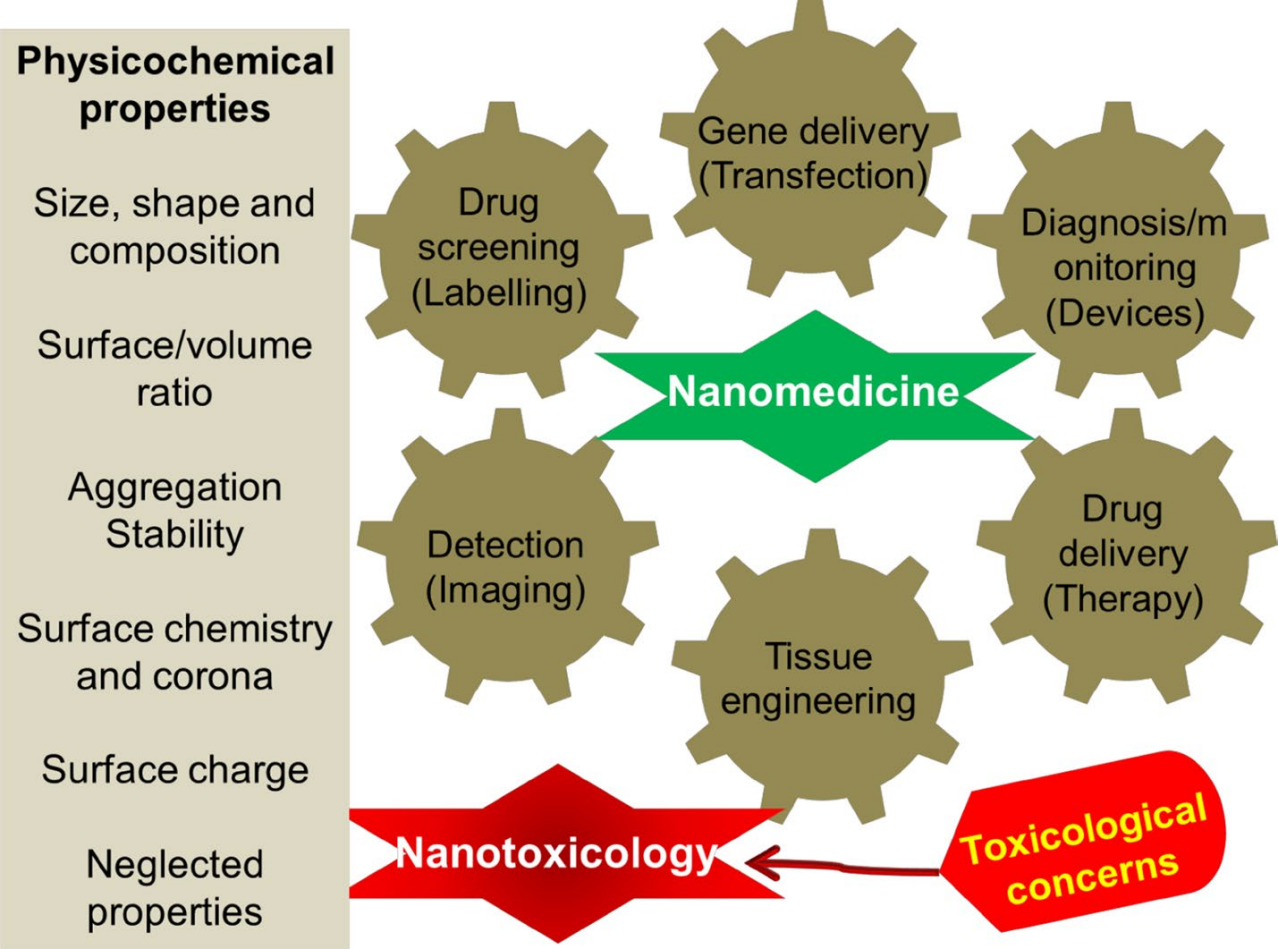
Schematic representation of physicochemical properties of nanomaterials which influences their biomedical applications; biomedical applications of nanomaterials (nanomedicine) and toxicological concerns (nanotoxicology)

Nanomaterials display exceptional physicochemical properties and can be exploited for various biomedical applications as illustrated in Fig. 3 due to their controlled size, high surface to volume ratio, differential shape, well-ordered composition, meticulous surface coatings or functional groups, solubility, specific hydrophilic or hydrophobic nature and aggregation. All these physicochemical parameters of nanomaterials either individually or cooperatively can affect the initial nano-bio interfacial interactions, adhesion of nanomaterials on cell membrane/surface, their cellular uptake or direct penetration inside the cells, and lastly nanomaterials communication with the cellular components; which ultimately translate into the bio-compatibility or -toxicity of these nanomaterials towards a specific biological entity leading to therapeutic or adversative effects [7, 52].

Moreover, different physicochemical belongings of nanomaterials have possibility to produce specific chemical atmosphere within the cells to encourage a pro-oxidant environment, initiating an imbalanced cellular energy system reliant on redox potential, thus leading to hostile biological consequences. Such hostile biological effects may range from the commencement of inflammatory pathways through to ultimate cell death [64]. Therefore, it is imperative to develop deeper insight into the physicochemical properties of nanomaterials and their biological aspects after nano-bio interactions to formulate better nanomaterials for future biomedical or pharmaceutical applications through nanomedicine. Likewise, the intrinsic physicochemical and surface properties of nanomaterials need to be carefully designed to accomplish specific biomedical applications as represented in Fig. 3 without any toxicological influence. The succeeding section discusses about various physicochemical properties of nanomaterials which may influences their potential biomedical/toxicological role, therefore need to be engineered prudently.

### Size, shape and composition

In the context of biomedical applications of engineered nanomaterials, the foremost distinctive feature is their size, which fall in-between individual atoms or molecules and corresponding bulk material. The reduced size of nanomaterials will not only provide an opportunity for increased uptake but also will build chances to interact with biological tissues to a greater magnitude to achieve desired type of selective biological action for therapeutic purposes [7]. Furthermore, in the recent times, it has been established that particle size is particularly domineering while other physicochemical parameters are controlled. To confirm this, systematic assessment of size-dependent biological profile and bio-distribution of three monodisperse drug-silica nano-conjugates of 20, 50 and 200 nm have been evaluated. This evaluation was performed through laboratory experiments in conjugation with mathematical modelling to establish the optimal size for the most effective antitumor drug delivery system. Through this study, it was revealed that the 50 nm sized drug-silica nanoconjugate particles had highest cancer tissue retention over time leading to deeper tissue penetration and effective internalization within the cancer cells along with slower clearance [65].

Additionally, nanomaterials are anticipated to cross biological obstacles, gaining entrance to the body and subsequently nano size may govern their kinetics, absorption, distribution, metabolism and excretion that would not be possible otherwise with the bulk material of akin composition [61, 66]. Well-defined gold and silver nanoparticles ranging within the 2–100 nm size and coated with antibodies have been reported to regulate the process of membrane receptor internalization leading to down regulate cellular expression level. This in turn alters the signalling processes and subsequently cellular responses, which are essential for basic cell functions. Furthermore, it has been demonstrated that nanoparticles of 40 and 50 nm size have greater effects not only due to their passive interaction with biological entities or cells but also due to their active engagement in mediating the molecular processes that are essential for regulating cellular activities [67]. In another study, ferromagnetic nanomaterials of three different sizes (300, 150 and 30 nm) were investigated for their intrinsic peroxidase-like catalytic activity. Interestingly, these nanomaterials displayed different levels of activity towards studied substrate, wherein smaller sized ferromagnetic nanomaterial revealed higher catalytic activity in the order of 30 > 150 > 300 nm; since smaller particles have greater surface-to-volume ratio to interact with substrates [68].

Likewise, theoretical traits propose that reduced particles size will have higher surface area, which may possibly promote interactions between the nanomaterials and the surface of biological entities, which may influence living organism adversely. In this perspective, size dependent toxicological consequences of various nanomaterials have been established by employing silver nanoparticles, palladium nanoparticles, single-walled carbon nanotubes and multi-walled carbon nanotubes toward bacterial cells. These studies show that smaller sized particles directly interact with bacterial cells leading to antagonistic effects confirming size-dependent toxicity [69–71]. Moreover, palladium nanoparticles illustrate that even the fine-scale of 1 nm dissimilarities can improve their antibacterial potential considerably and it will depend on the strains of the tested bacterial species [70].

In addition to size and type of material (composition), shape or morphology of a nanomaterial is another important characteristic feature at nanoscale level. However, only few investigations are focusing on the biomedical or toxicological relationship associated with this parameter alone. Nevertheless, in metallic nanomaterials various properties including electromagnetic, optical and catalytic properties are strongly influenced by their shapes [72–75]; consequently, it is believed that along with size of the nanoparticles, shape of nanomaterial also has substantial potential to influence nano-bio interfaces. In addition to leading cellular uptake, size of a material is key element that is related with the surface area for a specific mass-dosage. Generally, to the overall surface area, contribution of shape of the nanomaterials will be significant. For instance, an octagonal shaped nanomaterial will have different surface area compare to spheres of the equivalent size. Since surface atoms have a tendency to hold unsatisfied high energy bonds, the higher catalytic activity of nanomaterial with larger surface areas enhances its reactivity. Therefore, after effective entry within the cellular milieu these nanomaterials will have better probabilities compared to counterpart micron-sized particles to intermingle with biomolecules of cells, triggering direct cellular destruction and promoting oxidative stress [43, 76].

Recently, it was demonstrated that the shape of nanomaterial can impressively influence their rate of uptake by biological systems, wherein, spherical nanoparticles illustrate greater uptake over nanorods. Interestingly, internalization of nanorods was found to be dependent on their dimensions and high-aspect ratio rods were internalized considerably faster than low-aspect ratio nanorods [43]. Likewise, it has been reported that triangular nano-plates of silver displays higher antimicrobial activity, in comparison with spherical and rod-shaped silver nanoparticles against *Escherichia coli*. Furthermore, this study proposed that nano range size and the existence of (111) lattice plane combine to encourage antibacterial potential and nanoparticles commenced shape-dependent interaction with bacterial cells [77].

Likewise, recently a facile approach was employed by utilizing zwitterionic amino acids as reducing and stabilizing agents to obtain stable corona on metallic gold and silver nanoparticles along with different compositions. Antibacterial and in vitro peroxidase-like activities of composition controlled mono and bimetallic gold and silver nanoparticles confirmed and along with other physicochemical properties, composition of the nanomaterial has considerable effect on their biological actions. Interestingly, different antimicrobial profile was reported toward Gram positive and Gram negative bacterial strains, which was significantly influenced by the composition of nanomaterial [20, 78]. All these above discussed studies determine that nanomaterials should no longer be regarded as simple carriers for biomedical applications but need to be engineered for nanoscale delivery and therapeutic application keeping their nanotoxicity prospects in count, which is often neglected, if not overlooked.

### Surface/volume ratio and crystal planes

Along with size, surface to volume ratio is a significantly important physical property of any materials at nano scale level. It is imperative to recognize that number of surface molecules increases exponentially when the size of nanomaterial decreases below 100 nm; and, nanomaterial’s size and number of surface expressed molecules show inverse relationship [51, 79]. Nanomaterials size and surface area are important material characteristics from toxicological and biomedical applications perspective. As the size of nanomaterial decreases, its surface area increases. Increment in the surface area will allow a greater population of its atoms/molecules to be displayed on the surface of nanomaterial rather than its interior. For example, in a 30 nm sized nanomaterial, about 10 % of its molecules are expressed in the surface; while nanomaterials with 10 and 3 nm size will have 20 % and 50 % intensification in the surface expressed molecules, respectively. Since, number of atoms or molecules found on the surface of a nanomaterial are determinant of materials reactivity and biological profile; this will be fundamental for defining the chemical and biological property of nanomaterial [7].

Furthermore, reduction in size of material can construct irregular crystal planes, which can escalate the total number of structural defects and may disrupt ordered electronic configuration of the material, giving rise to altered electronic properties. All these physical changes can establish specific surface groups that could function as reactive sites such as hydrophilic or hydrophobic, catalytically active or passive etc. depending upon the chemical composition of the material [7, 79]. In other words, when taken together, it may be indicated that the greater surface area per mass compared with larger-sized particles of the same chemistry renders materials biochemically more active. This phenomenon of surface to volume ratio reflects the significance of chemical and biological activities of a nanomaterial since enhanced biological potential can be positive and desirable in terms of their antioxidant activity, carrier capacity for therapeutic purposes and penetration of cellular barriers (nanomedicine perspectives); or enhanced biological potential can be negative and undesirable such as toxicity, induction of oxidative stress or cellular dysfunction (nanotoxicology perspectives). In addition to above stated distinct positive and negative impacts, increased surface to volume ratio may have mix of both the properties at the same time [79]. Therefore, while engineering nanomaterials for any biomedical application point of view, special attention need to be paid on its surface to volume ratio and crystal planes, since these are predominantly responsible for various structural defects and surface properties which have often been neglected compare to other physicochemical properties.

### Aggregation, stability and protein corona

In addition to size, aggregation factor of nanomaterials need to be considered sensibly; however, this phenomenon has frequently been overlooked, if not than considered trivial for many biological and medical applications, which is misleading. Although numerous nanomaterials have been fabricated with a targeted size which may be ultra-small, yet these particles frequently form much larger colloidal aggregates. Stability of prepared nanomaterials against aggregation is always an essential concern before these nanomaterials employed for any biomedical application. Stability of synthesized nanomaterials depends on the pH of the medium in which the nanomaterials are dispersed and the electrolyte concentration in the solvent [80]. Stabilization of metal based nanomaterial in the solution can be accomplished by adding shielding or protecting agents which are required to avert agglomeration. Nanomaterials produced in solvents are usually unstable and incline to aggregate due to their higher free surface energy (result of their ultra-small size) [80, 81].

Nonetheless, aggregation phenomenon is suitable for aggregation-based immunoassays techniques. In this respect, metallic nanoparticles are considered the most relevant due to their optical properties. For instance, an aggregation-based simple, one step immunoassay has been developed by employing gold nanoparticles for anti-protein A. This extremely sensitive and specific assay was established based on the aggregation property of gold nanoparticles that were coated with protein antigens in the existence of their corresponding antibodies and monitored in terms of absorption change at 620 nm. Moreover, such gold nanoparticles centred aggregation assay is capable of analysing a variety of samples concurrently using microplate reader [82]. Furthermore, hybridization of target DNA in a cross-linking or non-cross-linking configuration is also possible by exploiting aggregation of DNA-functionalized gold nanoparticles, which opens up new possibilities for rapid, easy and reliable genetic diagnosis [83].

In addition to above conversed, it is vital to recognize that in an experimental set-up or physiological conditions many nanomaterials (especially metallic nanoparticles) have propensity to form agglomerate because of their inherent high reactive nature. Hence, when nanomaterials are introduced to living organism/cells in physiological environment or biological medium; it is expected that nanomaterials will construct aggregates rather existing as individual units; subsequently, the detected biological accomplishments will be outcome of agglomerated form of nanomaterial. For example, antibacterial activity of silver nanoparticles is size dependent, wherein smaller silver nanoparticles exhibit higher activity on the basis of equivalent silver mass content. Conversely, silver nanoparticles have inclination to aggregate in media due to high electrolyte content, causing loss of their antibacterial effectiveness. However, complexion of silver nanoparticles with stabilizing agents by surface modification or surface coatings can stabilize them against aggregation, leading to retention of their antibacterial potential [84]. In order to improve antibacterial prospects by regulating aggregation of nanomaterials, hybrid composites of nano silver-silica (Ag–SiO_2_) and nano copper-silica (Cu–SiO_2_) were prepared, wherein silver nanoparticles or copper nanoparticles were uniformly distributed on the surface of silica nanoparticles deprived of any emblem of aggregation and demonstrated higher antibacterial capacities due to the lack of aggregation [85, 86]. Aggregation property of nanomaterial can be utilised for immunoassays, diagnosis, biosensing, antimicrobial and other applications. Therefore, nanomaterials need to be designed rationally while utilizing aggregation phenomenon for biomedical applications with their potential stability and toxicity in count.

Interestingly, nanomaterials, in general will be taken-up via endocytosis process, during which they are exposed to highly varying pH conditions ranging from 7.4 (extracellular medium), 5.5 (late endosomes), to 4.5 (lysosomes). Therefore, the chemical strength of the nanomaterials exposed to the disintegrative endosomal environment gaining increasing consideration. In this context, it is further imperative to notice that in addition to acidic pH, lysosomes possess high levels of hydrolytic bio-catalysis that has potential to degrade any nanomaterials completely or to their surface corona, which is required for particle stability. Besides, it has been reported that after endosomal uptake of nanomaterials, conjugated or non-specifically bound proteins degrades rapidly by a low-specific protease Cathepsin L, leading to significant loss of function of bio-conjugated particles. In particular, for nanomaterials that were encompassing intracellular targeting molecules or pharmaceutical active drugs such effects can have noteworthy consequences. Likewise, mostly biologically relevant moieties will be degraded more easily; the nanomaterials can be stripped from their surface corona resulting in different physicochemical properties such as intra-endosomal aggregation [87–89].

On the contrary, proteins bind to the nanoparticles in biological fluids leading to generating surface coating on a nanomaterial known as the protein corona. This protein corona considerably affects the interaction of the nanomaterials with biological systems. In highly dynamic physiological systems it is imperative to understand the formation and development of protein-corona and its biological relevancy prior to employing such materials for biomedical applications. In this viewpoint, by using silica and polystyrene nanoparticles of various size and surface functionalization in human plasma, corona formation has been studied. This study has revealed rapid material-specific corona formation of almost 300 different proteins. Furthermore, it has been established that though the composition of specific corona did not differ considerably over the time but the amount of bound protein changed significantly. The properties of the biomolecules derived surface corona can be directly linked to its biological impacts. Therefore, critical assessment and basic knowledge of such nano-bio interfacial interactions became imperative in terms of rates, affinities and stoichiometries of protein association with, and dissociation from respective nanomaterial. Proteins associated on the surface of a nanomaterial, amount and arrangement of the proteins on the surface can play a central role in an in vivo response [90]. It has already been established that the rapid corona formation affects haemolysis, thrombocyte activation, nanomaterial uptake and endothelial cell death at an early exposure [91–93]. Interestingly, amendment in secondary structure of protein and consequent changes in its activity upon binding to nanomaterials surface may have disadvantage and it may be a potential source of nanotoxicity. However, such functional nanomaterials can be utilized towards promising applications of nanoparticles in increasing protein stability toward enzyme degradation and increasing enzymes activity via immobilization at surfaces [94]. From the above discussion, it is apparent that alongside aggregation and stability, formation of protein corona is an imperative physicochemical property need to be considered carefully due to its influential role at nano-bio interface.

### Surface functionalization/chemistry and exterior corona

In order to retain biomedical potential of nanomaterials, it is vital to control their aggregation characteristic and develop specific chemistry or surface corona on nanomaterials exterior, which can be achieved by their surface coatings or functionalization. In addition to controlling aggregation, tailored surface corona or functionalization of nanomaterials may generate different interesting opportunities to develop efficient nano-agents in highly controlled fashion for biomedical applications [25, 26]. In this context, design and development of surface-modification schemes for silica nanoparticles have been suggested wherein an optimum balance of inert and active surface functional groups was strategically attained to reduce particle aggregation and their nonspecific binding. Where, silica nanoparticles were primed in a water-in-oil microemulsion followed by co-hydrolysis with tetraethyl orthosilicate (TEOS) and different organosilane reagents in order to develop various surface modifications. Moreover, it has been demonstrated that by employing suitable surface-modification stratagem, fluorescent dye-doped silica nanoparticles can be readily conjugated with biological molecules for DNA chip or other type of bio-analytical applications as sensitive, reproducible and fluorescent labels [95]. Likewise, other nanomaterials such as magnetic iron oxide nanoparticles, zinc oxide, carbon nanotubes, gold nanoparticles, silver nanoparticles and many more can be functionalized by small molecule ligands, polymers and biomolecules [9, 18, 25, 26, 96–98].

**Fig. 4:**
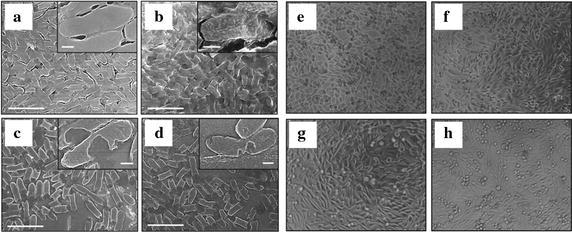
SEM micrographs of *E. coli* and phase contrast micrographs of human PC3 epithelial cells (**a**, **e**) before and (**b**–**d**, **f**–**h**) after treatment with AgNPs^Tyr^, AgNPs^Tyr@PTA^ and AgNPs^Tyr@PMA^, respectively (adopted from Ref. [34])

Recently, a new synthetic scheme has been established wherein gold and silver nanoparticles were surface functionalized by creating stable surface corona of biologically-active polyoxometalates (POMs) and precise surface chemistry [25, 26]. This functionalization was accomplished by employing zwitterionic amino acid tyrosine as a pH-switchable reducing and capping agent around silver nanoparticles. Furthermore, significant improvement in antibacterial profile of both gold and silver nanoparticles was reported due to enhancement in degree of physical destruction, as illustrated for silver nanoparticles in Fig. 4a–d [25, 26]. Interestingly, reported silver nanoparticles exhibited significant antibacterial potential toward both tested Gram negative and positive bacterial strains with similar toxicity pattern. Nevertheless, further investigation on PC-3 epithelial cells revealed that these functionalized silver nanoparticles do not have any significant cytotoxicity or physical damage toward mammalian cells as shown in Fig. 4e–h. Although, authors could not provide full explanation for the discriminating toxicity of silver nanoparticles towards the tested Gram bacterial strains and PC-3 epithelial cells however, with reference to Clement and Jarrett [99] report it was established that the toxicity of silver to human cells is substantially lower than to bacteria. Moreover, most widely documented usages of silver are prophylactic treatment of burns and water disinfection. Alike outcomes have been confirmed with the biosynthesized silver nanoparticles, which displayed admirable antibacterial efficacy toward both Gram positive and negative bacteria but exposed good cytocompatibility with mammalian cells [100]. Furthermore, in vitro toxicity of silver nanoparticles at non-cytotoxic doses has been evaluated in human hepatoma cell line and HepG2 by various assays, wherein it was revealed that silver nanoparticles accelerate cell proliferation at low doses (<0.5 mg/L) due to stimulation of genes associated with cell cycle progression. Contrariwise, noteworthy cytotoxicity at higher doses (>1.0 mg/L) was reported due to abundant abnormal morphological changes. Further, in this study it was established that both silver nanoparticles and leaching of Ag^+^ ions from nanoparticle contribute to the toxic effects [101]. Therefore, thorough understanding of leaching behaviour of Ag^+^ ions from particle, their kinetics and toxicity of silver nanoparticles yet need to be established in the context of their underlying medical debate for the safe use of silver based materials. Nevertheless, based on the available knowledge it can be proposed that such engineered nanomaterials can be used for specific antimicrobial targeting without any considerable damage to mammalian cells at lower concentrations [25].

**Fig. 5:**
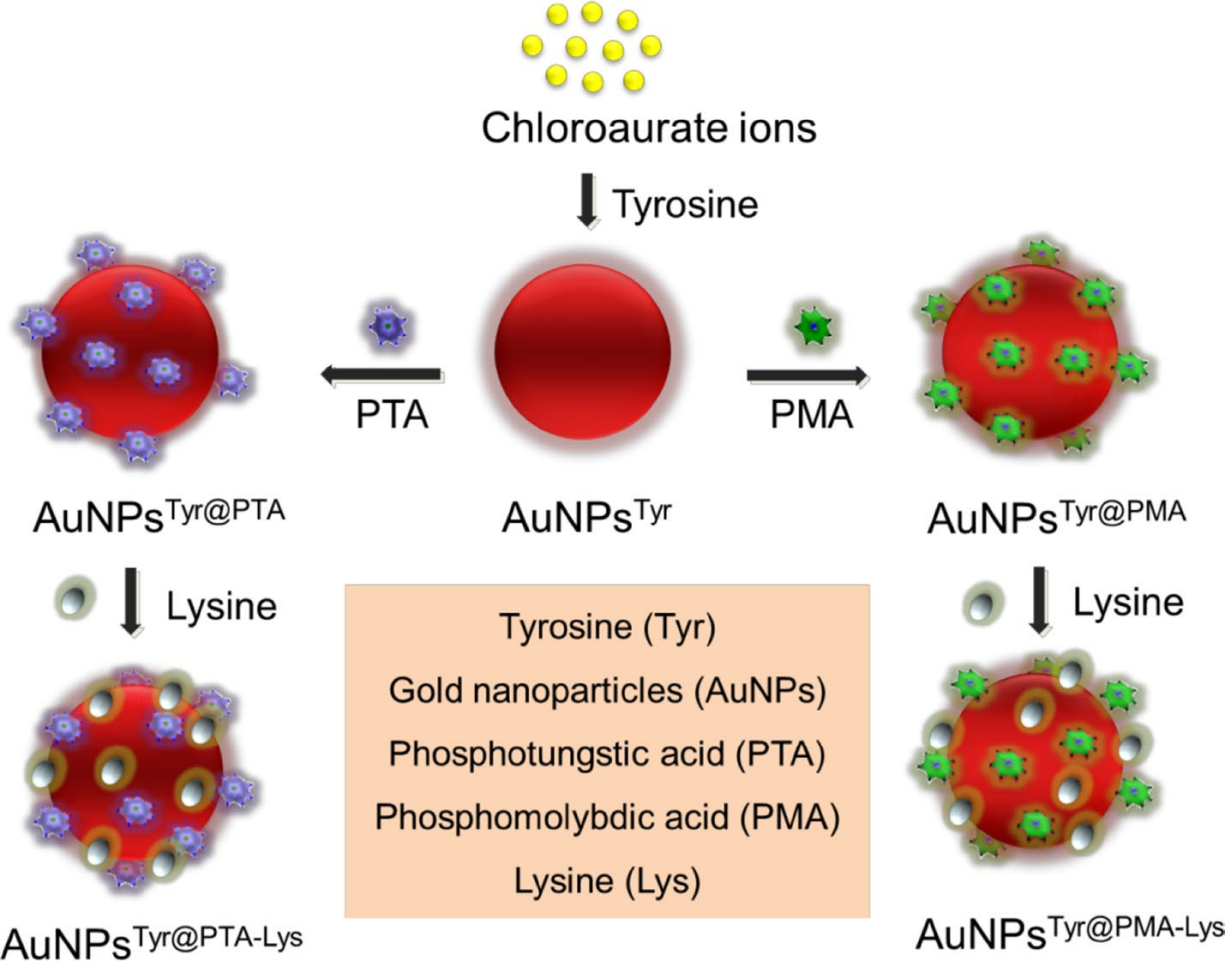
Schematic representation of tyrosine-mediated synthesis of gold nanoparticles, followed by their sequential surface functionalization with PTA or PMA and lysine (adopted from Ref. [33])

Furthermore, sequential surface functionalization approach was verified in the case of gold nanoparticles by using cationic amino acid lysine in the outermost shell as exemplified in Fig. 5, to assist these gold nanomaterials in directing toward negatively charged bacterial cells. This research revealed that gold nanoparticles, which are considered highly biocompatible in nature, can be regulated to be a strong antimicrobial agent by fine-tuning their surface functionalization in a controllable manner [26]. These investigations recommend that facile tailorability of nanomaterials surfaces may play a substantial role in controlling their biological activities.

From the above discussion, it is clear that tuneable surface functionalities of various nanomaterials provide versatile scaffolds for a variety of biomedical applications. For example, appropriate control of surface properties can exploit therapeutic efficacy whereas it can reduce hostile side effects. In addition to this, attentive choice of nanomaterial functionalization/coating can decrease the adverse influence on the environment [102]. However, several essential features of nanomaterials surface functionalization need to be addressed during translation from experimental triumph to clinical preparation. For rational design of nanomaterials, surface modifications should be fabricated to provide biomimetic properties like stability in complex biological media, non-cytotoxicity and specificity toward a particular biological entity. Such acquaintance of nanomaterials surface functionalization dependent biological activities may have impact on designing effective therapeutic nanomaterials for diagnosis and treatment of diseases [98]. From the above discussion, it can be highlighted that the nanomaterial toxicity and biological applicability are strongly governed by their surface functionalization and exterior corona, which need to be engineered with extreme carefulness for any biomedical application for specificity and non-cytotoxicity.

### Surface charge

Alongside surface corona and surface chemistry, surface charge is a distinguishing physicochemical property of a nanomaterial. Surface charge of a particular nanomaterial has potential to govern its biomedical and toxicological actions and it is critical for providing insight of nano-bio interaction under different experimental set-ups. In addition to ionic strength and solution pH, surface charge has significant influence for the progress of aggregation in aqueous milieu. Furthermore, it plays fundamental role in governing initial electrostatic interaction at nano-bio interface and positive surface charged nanomaterials have been reported for toxicity on living organisms. Peripheral surface layers can convey selective charge on nanomaterials providing them stability as discussed earlier while guiding their surface chemistry. Moreover, the cellular entry of a nanomaterial definitely depends on its surface charge and toxic effects of positively charged nanomaterial have widely been explored, but it was not observed when the same material coated with negatively charged functional groups [103, 104].

It has also been reported that nanomaterials with cationic surface charge are more likely to intermingle with the genetic material triggering genotoxicity due to negative charge on DNA. On contrary, if surface charge on a nanomaterial is alike charge of the cell membranes, this may induce repulsion and prevent nanomaterial-cell contact. In general, cell membranes possess negative charge, therefore, it is believed that nanomaterials with negative surface charge may internalize slower compared to their positively surface charged counterparts. Furthermore, contemporary research suggest that positive surface charge bearing nanomaterials are primarily being internalized through clathrin-mediated endocytosis (CME) including chitosan, PLGA modified with PLL, amino group-modified SNTs etc. However, some exception have been documented wherein multiple pathways including caveolae-mediated endocytosis were observed for internalization of strong cationic surface charged nanomaterials [105]. The impact of surface charge on cellular uptake and in vitro cytotoxicity of mesoporous silica nanoparticles in human mesenchymal stem cells (hMSCs) has been evaluated; wherein, it was revealed that the mesoporous silica nanoparticles uptake by hMSCs can be regulated by a threshold of positive surface charge. In addition to this, further it was proposed that the inflection of surface charge on mesoporous silica nanoparticles uptake is specific to cell type [106].

Moreover, critical role of surface charge of gold nanoparticles in modulating membrane potential of different malignant and non-malignant cell types followed by their downstream intracellular events was established in recent times; which revealed a novel mechanism for cell-nanoparticle interactions and gold nanoparticles uptake. Positively charged gold nanoparticles were taken-up intracellularly based on the membrane potential and generate membrane depolarization. This action improved [Ca^2+^]_intracellular_ by increasing Ca^2+^ influx and inducing release of intracellular Ca^2+^ stores via endoplasmic reticulum through IP_3_ receptor channels. All these variations can result in higher apoptosis and lower cell proliferation, subjected to cell type. Added modulation of cell apoptosis and proliferation may involve direct nanoparticle effects on intracellular signalling mechanisms [107]. All such studies with reference to surface charge of various nanomaterials are expected to help in developing understanding of various biological events of cell-nanoparticle contacts, which will support in expedite the engineering of nanoparticles for specific intracellular targets for therapeutic applications with controlled toxicological perspectives.

### External and neglected properties

In addition to various inherent physicochemical characteristics as discussed in previous sections, external influences may also have noteworthy consequence on biomedical or toxicological belongings of nanomaterials. For example, in the presence of light, photosensitive nanomaterials will be capable of producing higher amount of reactive oxygen species (ROS) which will have considerable toxicological effects. In this context, three photosensitive nanomaterials, titanium dioxide, silicon dioxide and zinc oxide have been assessed to possess toxicity with varying degrees toward Gram positive and Gram negative bacterial strains in water suspensions by particle concentration dependent manner. This research claims that the stimulus of light had significant influence under most of the examined experimental conditions which is possibly related with its role in motivating production of ROS [108].

Furthermore, purity of any nanomaterial is one of the most important characteristic, which need to be considered for its biomedical or toxicological role. However, it has often been neglected, which needs to be considered for its active role in therapeutics or otherwise. Existence of residual contaminating foreign metals, unreduced metal ions, chemicals or other agents (from the precursor material used for nanomaterials synthesis) may actually be responsible for noxious actions rather than the actual nanomaterials itself and the quantity of contaminating materials are fully dependent upon the synthesis method used. Currently, numerous post-production nanomaterials processing methods are known to remove most of these precursor metal catalysts and chemical agents from nanomaterials; however, such purified nanomaterials may still have some amount of remaining substances. Therefore, the effects of such chemical impurities, residual metals and presence of counter ions on both potential biomedical and deleterious effects cannot be overlooked. Hydrophobicity/hydrophilicity, electron transfer capability, surface smoothness/roughness/defects, oxidizability of nanomaterials in physiological conditions and counter ion effects are other essential physicochemical parameters of diverse nano objects that need to be considered to control their toxic potential while engineering nanomaterials for their biomedical applications. For instance, one of the recent studies used multiparametric methodology to understand high-content imaging coupled with gene expression analysis on fundamental pathways for evaluating cell-nanomaterial interactions. By employing this approach the effect of the surface charge and hydrophobicity of gold nanoparticles on cell-material interactions were parametrically evaluated followed by their validation through biochemical assays. Interestingly, the data evidently divulge that while surface hydrophobicity of nanomaterial does not essentially affect cellular uptake levels, nevertheless increased surface hydrophobicity was found to be associated with higher cell membrane damage and induction of autophagy, which had greater influence than the effect of surface charge ranging between −50 and +20 mV [109]. In another study, it has been confirmed that hydrophobic and hydrophilic graphene can differentially influence nano-bio interactions and their toxicity profile. Comparison between, highly hydrophobic pristine graphene and carboxyl functionalized hydrophilic graphene with monkey renal cells have revealed large accumulation of hydrophobic graphene on the cell membrane inducing intracellular reactive oxygen species (ROS) stress leading to apoptosis, whereas functionalized hydrophilic graphene was internalized by the cells without causing any toxicity. These results were evident from confocal microscopy and cell function assays confirming significant importance of surface pacification to control strong hydrophobic interaction associated with toxicity effects of graphene through carboxyl functionalization. However, it is imperative to state that graphene is a non-biodegradable material with higher cellular internalization capacity. Therefore, the potential long-term hostile effects of functionalized hydrophilic graphene need to be explored yet to realize their full biomedical capabilities [110]. From the discussion, it can be clinched that the controlled experimental conditions and suitable functionalization may provide comparability across studies. This is imperative for reliable illustration of nanomaterial structure–activity correlations, which is prerequisite for the potential application of nanoparticles in medicine.

## Conclusion

Nanotechnology has significant potential to influence field of biology and medicine due to nanoscale size of basic biological entities and it is gaining considerable attention in terms of nanomedicine. However, toxicological perspectives of engineered nanomaterials are poorly understood or rather unclear, which is limiting full potential of nanomedicine. Therefore, the often ignored toxicological concerns of engineered nanomaterials need urgent attention and it is essential to carry out fundamental research to address these issues. Moreover, the future of nanomedicine will depend on rational engineering of various nanomaterials with controlled physicochemical properties to dictate their interactions in anticipated manner with biological systems for biomedical applications. Additionally, detailed and thorough understanding of nano-bio interactions will be required to discover favourable physicochemical characteristics of various nanomaterials, which may render them more responsive toward inner biological environment for therapeutic benefits without any toxic impact.
